# Beyond COX-1: the effects of aspirin on platelet biology and potential mechanisms of chemoprevention

**DOI:** 10.1007/s10555-017-9675-z

**Published:** 2017-07-31

**Authors:** Argentina Ornelas, Niki Zacharias-Millward, David G. Menter, Jennifer S. Davis, Lenard Lichtenberger, David Hawke, Ernest Hawk, Eduardo Vilar, Pratip Bhattacharya, Steven Millward

**Affiliations:** 1 Department of Cancer Systems Imaging, Division of Diagnostic Imaging, The University of Texas MD Anderson Cancer Center, Houston, TX USA; 2 Department of Gastrointestinal (GI) Medical Oncology, Division of Cancer Medicine, The University of Texas MD Anderson Cancer Center, Houston, TX USA; 3 Department of Epidemiology, Division of Cancer Prevention and Population Sciences, The University of Texas MD Anderson Cancer Center, Houston, TX USA; 4 McGovern Medical School, Department of Integrative Biology and Pharmacology, The University of Texas Health Science Center at Houston, Houston, TX USA; 5 Department of Systems Biology, Proteomics and Metabolomics Facility, The University of Texas MD Anderson Cancer Center, Houston, TX USA; 6 Department of Clinical Cancer Prevention, Division of OVP, Cancer Prevention and Population Sciences, The University of Texas MD Anderson Cancer Center, Houston, TX USA

**Keywords:** Platelets, Aspirin, Cyclooxygenase-1, Cyclooxygenase-2, Chemoprevention, Acetylome

## Abstract

After more than a century, aspirin remains one of the most commonly used drugs in western medicine. Although mainly used for its anti-thrombotic, anti-pyretic, and analgesic properties, a multitude of clinical studies have provided convincing evidence that regular, low-dose aspirin use dramatically lowers the risk of cancer. These observations coincide with recent studies showing a functional relationship between platelets and tumors, suggesting that aspirin’s chemopreventive properties may result, in part, from direct modulation of platelet biology and biochemistry. Here, we present a review of the biochemistry and pharmacology of aspirin with particular emphasis on its cyclooxygenase-dependent and cyclooxygenase-independent effects in platelets. We also correlate the results of proteomic-based studies of aspirin acetylation in eukaryotic cells with recent developments in platelet proteomics to identify non-cyclooxygenase targets of aspirin-mediated acetylation in platelets that may play a role in its chemopreventive mechanism.

## Introduction

The use of aspirin derivatives dates back thousands of years. Salicylates were initially derived from plant extractions; salicylic acid in particular was isolated from the Willow tree. In 1897, Felix Hoffman developed a method to retain the analgesic and antipyretic properties of salicylic acid while decreasing the side effects associated with prolonged administration. This was done by acetylating the phenolic hydroxyl group of salicylic acid to form acetylsalicylic acid (aspirin). By the early 1900s, the therapeutic benefits of aspirin (and its salicylate) were widely recognized, and over time, other drugs were developed that had the same anti-inflammatory, analgesic, and anti-pyretic activities. These include antipyrine, phenacetin, ibuprofen, and naproxen, which target both cyclooxygenase 1 and 2 (COX-1 and COX-2), and more recently celecoxib and rofecoxib, which are predominantly COX-2 specific. These are broadly grouped into a category of drugs known as non-steroidal anti-inflammatory drugs (NSAIDs). NSAIDs are key medications for the treatment of pain, fever, and inflammation. In addition to its initial use as an analgesic and anti-pyretic, aspirin is commonly used today at low daily dosages to prevent cardiovascular disease (CVD). Recent epidemiological studies have revealed a dramatically reduced incidence of cancer in individuals taking daily low-dose aspirin [1–7], suggesting that aspirin has a powerful chemopreventive effect as well [1, 2, 7].

Aspirin, like the vast majority of NSAIDs, is thought to exert its anti-inflammatory effects through inhibition of cyclooxygenase enzymes (COX enzymes) that regulate the production of prostaglandins. COX-1 is expressed constitutively in most tissues and regulates basal levels of prostaglandins which control platelet activation and protect the lining of the gastrointestinal tract [8]. In contrast, COX-2 is inducible and responsible for releasing prostaglandins after an infection, injury, or in cancer development. Prostaglandins mediate a number of biological effects including the induction of an inflammatory immune response. By inhibiting prostaglandin biosynthesis, particularly the precursor prostanoids PGG_2_ and PGH_2_, aspirin acts to blunt a variety of pro-inflammatory responses, including the canonical inflammatory response [9–11], production of a defensive mucosal lining [12], and platelet aggregation [13, 14]. In addition to modulating the inflammatory response, aspirin has a dramatic effect on the biochemistry and physiological function of platelets.

Platelets, which are small anucleate cell fragments derived from megakaryocytes, play a key role in the clotting response. There is also a growing body of evidence linking platelet and platelet functions to tumorigenesis and metastasis. Given the robust inhibition of platelet function by aspirin and the known epidemiological link between aspirin use and cancer prevention [2, 7], it is of interest to revisit the effect of aspirin on platelet biochemistry in the context of oncology. This review will focus on the chemistry of aspirin and its effect on the biochemistry and biological functions of platelets. While many of these effects are mediated directly or indirectly by COX-1 inhibition, other non-COX-dependent mechanisms will also be discussed. We will begin with a brief introduction to the chemistry and pharmacology of aspirin along with a brief survey of the clinical data supporting its chemopreventive role. We will then discuss platelet biology and the effects of aspirin-mediated cyclooxygenase inhibition on prostaglandin synthesis and the on prostaglandin synthesis and the platelet aggregation cascade. We will examine recent studies on non-cyclooxygenase targets of aspirin and their potential role in platelet biochemistry. We will conclude with a discussion on the potential roles of platelets in cancer chemoprevention and how these effects could be modulated by aspirin.

## The chemistry and pharmacology of aspirin

**Fig. 1:**
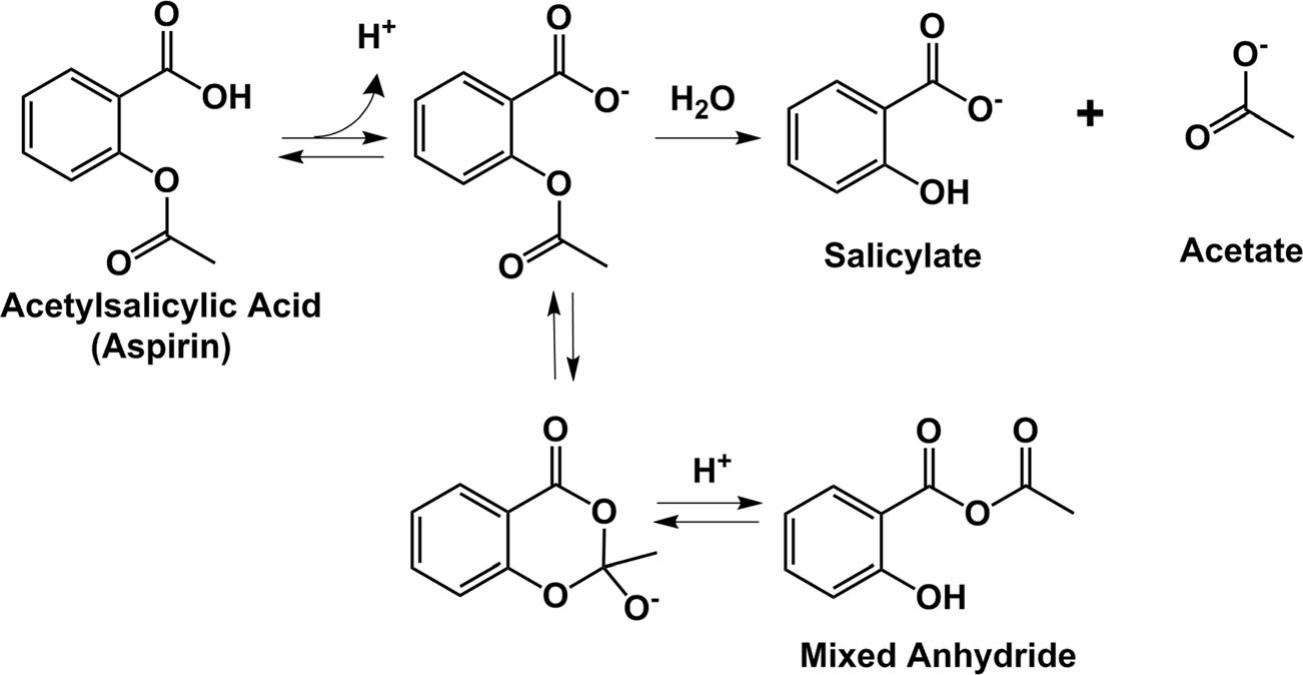
The Chemistry of Aspirin Under Basic Conditions. Deprotonation of the carboxylic acid results in the formation of the aspirin anion which abstracts a proton from water to generate a nucleophilic hydroxide anion. The negatively charged hydroxide attacks the carbonyl carbon of the acetate group resulting in hydrolysis of aspirin into salicylate and acetate (general base catalysis) [15, 16]. Recent work also suggests that a mixed anhydride can be formed under basic conditions through a hemiorthoester anion intermediate [18] although the contribution of this intermediate to the mechanism of hydrolysis is unknown

Aspirin is an O-acetyl derivative of salicylic acid (ASA—acetylsalicylic acid) and its dominant mechanism of action is believed to be through the transfer of this acetyl group to (−OH) and amino (−NH_2_) functionalities present in biological macromolecules. The acyl ester group is also unstable under basic conditions, and its hydrolysis to acetate is believed to proceed by a general base-assisted mechanism as described previously [15, 16]. More recent computational studies have suggested an n→π* interaction between the aromatic carboxylic acid and the carbonyl carbon of the acetate group [17]. This is consistent with a nuclear magnetic resonance spectroscopy (NMR) study [18], which posits the formation of a cyclic hemiorthoester under basic conditions which can rearrange to give either the parent aspirin anion or a mixed anhydride (Fig. 1). Although the prevalence and role of the mixed anhydride in the biochemistry of aspirin has yet to be determined, the broad scope of anhydride reactivity may help to explain promiscuous acetylation activity of aspirin in biological systems [19, 20]. Interestingly, it has also been shown that the mixed anhydride can react with the primary amino group of glycine in organic solvents to form N-salicyloylglycine, suggesting a second class of aspirin-mediated protein modifications [21].

The non-selectivity of aspirin-mediated acetylation was demonstrated by Richard Farr and co-workers in 1968 [22]. In these experiments, aspirin labeled with ^14^C at the acetyl carbonyl carbon was incubated with a series of blood proteins as well as common enzymes and nucleic acids. Following dialysis, substantial radiolabeling of albumin, immunoglobulins, α-macroglobulin, and other enzymes was observed. More recent mass spectrometry-based studies have validated this initial finding and the list of proteins acetylated by aspirin has grown to include histones, IKKβ (I-kappa-β-kinase beta), and many others [23–33]. At high concentrations (micromolar to millimolar), aspirin has been shown to react with nucleophilic groups on proteins resulting in irreversible acetylation. These include the functional groups of the residues lysine (−NH_2_), arginine (−NH_2_), serine (−OH), threonine (−OH), tyrosine (−OH), and cysteine (−SH).

The synthesis of aspirin is highly straightforward. Aspirin can be synthesized under acidic or basic conditions using acetyl chloride or acetic anhydride in the presence of salicylic acid [34, 35]. Synthesis of ^13^C- or ^14^C-labeled aspirin has also facilitated the real-time analysis of acetylation of ubiquitin [28], hemoglobin [36], and human serum albumin [34].

## Pharmacokinetics and pharmacodynamics of aspirin

Inhibition of COX-1 and COX-2 activity by aspirin is attributed to the covalent modification of active site serine residues (Ser 530 in COX-1 and Ser 516 in COX-2) [37, 38]. Acetylation of these side-chain hydroxyl groups results in irreversible inhibition through steric blockade of the active site. This effect can be recapitulated by acetic anhydride, which acetylates nucleophilic groups, albeit at a much higher rate than aspirin [23, 39].

While site-selective acetylation of COX-1 and COX-2 is thought to be driven in part through molecular recognition of the benzoic acid functionality of aspirin, the “non-specific” acetylation activity of aspirin is thought to be driven largely by the chemical environment. For example, in the highly acidic environment of the gastric mucosa, (pH = 2–3), the carboxylic acid (pK_a_ = 3.5) exists mainly in the protonated state which is predicted to reduce the rate of hydrolysis. In contrast, the alkaline environment found in the gastroduodenum (pH = 8.0) results in deprotonation of the carboxylate group and an increased rate of both hydrolysis and transacetylation [30]. In addition to environmental pH, the aromatic ring and carboxylic acid also play important roles in aspirin’s reactivity and stability. These effects are mediated through hydrogen bonding of the carboxylic acid and/or the free hydroxyl, or through *π* − *π* stacking interactions with the aromatic ring. The stabilization of the salicylic acid moiety has also been shown to be important for efficient acetylation of free nucleophilic residues on protein surfaces [37, 38].

Aspirin is readily absorbed in the acidic environment of the gastric mucosa. At this interface, aspirin can readily inhibit the biosynthesis of prostaglandins that are associated with protection of the stomach lining [12, 31, 32]. Absorption in the stomach lining is facilitated by the molecule’s net neutral charge resulting from protonation of the carboxylic acid at low pH. As predicted by mechanistic studies, very little, if any, aspirin undergoes spontaneous hydrolysis at this pH. In addition, at lower pH, it would be expected that the protonation of the carboxylate would prevent the intramolecular rearrangement of aspirin to the acetylsalicylic acetic anhydride [21]. As aspirin moves from the highly acidic environment of the stomach to the nearly neutral pH of the duodenum (pH 7–8) and the small intestine (pH 7.3), deprotonation of the aromatic carboxylic acid is favored resulting in a net negative charge.

The half-life of aspirin in the bloodstream was previously shown to be 13–19 min with a non-enzymatic hydrolysis rate of 0.023 min^−1^ at 37 °C in individuals given a single oral administration of aspirin. Approximately 70% of aspirin reaches the peripheral circulation intact with maximum serum concentrations observed at 25 min after administration. After entering the bloodstream, aspirin undergoes enzymatic hydrolysis to yield acetate and salicylic acid. The major enzymes hydrolyzing aspirin in plasma are believed to be cholinesterases [40], which is supported by the observed decrease in hydrolysis in the presence of anticholinesterase inhibitors [41]. Most recently, acetylhydrolase I, an intracellular erythrocyte platelet-activating factor, has been characterized as the major aspirin hydrolase of human blood [42]. In the liver, carboxyesterases are believed to carry out this role.

In the blood stream, platelet uptake of aspirin is driven by concentration-dependent passive diffusion [43]. *In vitro* studies have shown that 20% of soluble aspirin is taken up in platelets although only 0.05% undergoes acetyl transfer to cellular proteins as measured by SDS gel electrophoresis [44]. Intravenous aspirin has a distribution half-life of about 3 min and inhibits prostaglandin biosynthesis within 5 min of administration, reflecting the rapid onset of inhibition compared to oral dosing [45]. Enteric coated aspirin has been employed to decrease the bleeding effects in the gastrointestinal tract. This formulation typically increases the rate of absorption of aspirin and delays its metabolism and activity. Another study showed that enteric-coated aspirin results in both delayed onset of anti-platelet activity and a loss of aspirin bioavailability due to hydrolysis [46]. Recent studies by Lichtenberger et al. demonstrated that aspirin could enter the lymph fluid directly when administered intragastrically or intraduodenally, potentially increasing its pharmacologic activity as a chemopreventive agent for colorectal cancer [47].

The distribution of aspirin is further enhanced by binding to human serum albumin [48, 49]. Human serum albumin is the most abundant protein found in blood and is often used as a plasma shuttle for steroids, hormones, and other small molecules. Binding studies suggest a conformational change in albumin upon acetylation that can influence transport and metabolism of other critical metabolites and drugs. For example, aspirin-induced acetylation of albumin can inhibit glucose binding [50], while increasing the binding of other molecules, as observed with the increased affinity of acetylated albumin for the marker anion acetrizoate [51]. Aspirin pharmacokinetics and pharmacodynamic are also influenced by the interaction of other metabolites and serum albumin [49]. However, aspirin acetylation of serum albumin likely inhibits the binding of other metabolites commonly transported by albumin. *In vitro* studies have shown serum albumin binding and acetylation is dependent upon fatty acid binding [52], pH [53], and temperature [54].

**Fig. 2:**
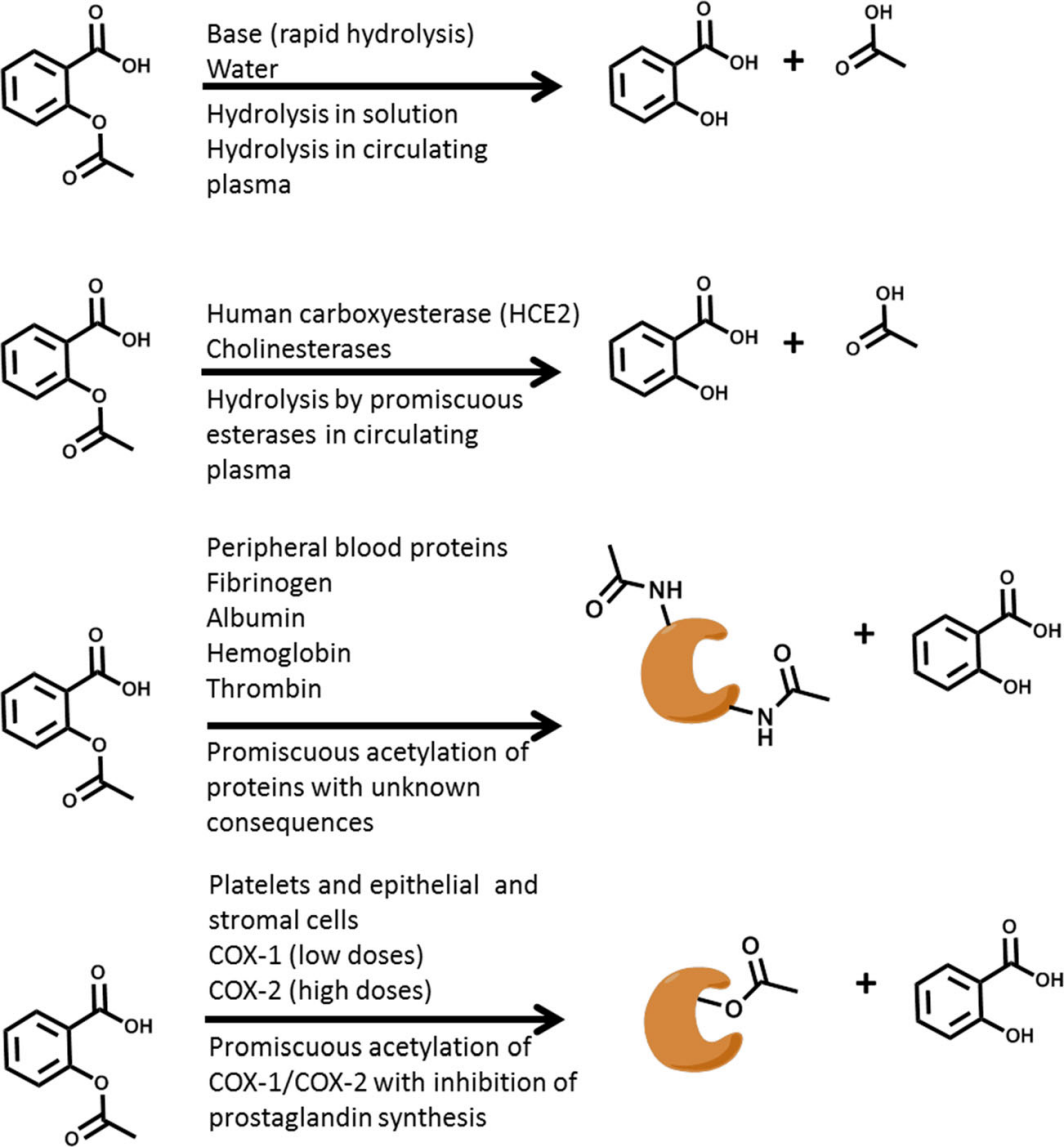
Reactivity of aspirin in different biological environments of proteins

The major route of elimination of aspirin is through its hydrolyzed product salicylic acid**.** Salicylic acid is cleared from circulation *via* the kidneys with a serum half-life of approximately 2 h [55]. A summary of the most common reactions of aspirin in biological systems are summarized in Fig. 2.

## Clinical trials to evaluate the chemopreventive effects of aspirin

Ishikawa et al. analyzed 51 randomized controlled trials (RCTs) and the cumulative evidence strongly supports the hypothesis that daily use of aspirin results in the prevention of cardiovascular disease (CVD), as well as a reduction in cancer-associated mortality [3]. Six other trials also showed reduced gastrointestinal cancer incidence after 3 years of aspirin use (OR, 0.76, 95% CI, 0.66–0.88; *p* = 0.0003) with statistically significant reductions also observed for esophagus, colon, and lung cancers. All cancer preventive, incidence, mortality, and metastasis effects were more pronounced with increased duration of the scheduled trial treatment. Importantly, all fatalities from major extra-cranial bleeds were lower in aspirin compared to controls, with a favorable risk/benefit ratio. Other studies have also demonstrated reductions in cancer incidence [56], particularly for cancers of the gastrointestinal tract, along with cancer-related [57–59] and all-cause mortality [57, 58, 60]. Other evidence that NSAIDs and aspirin prevent cancer was shown in multiple clinical trials involving short-term aspirin use at varying doses that reduced colorectal adenomas. For patients with a previous history of colorectal cancer (CRC) [61] or colorectal adenomas, [62, 63], those taking aspirin showed fewer new adenomas compared to no-aspirin controls. In The Colorectal Adenoma/Carcinoma Prevention Programme (CAPP) trial, long-term use of aspirin in a cohort with hereditary CRC (Lynch Syndrome) patients revealed an HR of 0.63 (95% CI, 0.35–1.13; *p*=0.12) [5].

Looking forward, in the ASPirin Intervention for the REDuction of colorectal cancer risk (ASPIRED) trial, the effects of aspirin will be examined using various biomarker endpoints [64]. ASPIRED is designed as a prospective, double-blind, multidose, placebo-controlled, biomarker clinical trial of aspirin use in a cohort of patients previously diagnosed with colorectal adenoma. Subjects (*n* = 180) will be randomized to low-dose (81 mg/day) or standard-dose (325 mg/day) aspirin or placebo. These individuals will give lifestyle, dietary, and biometric data. They will also provide urine, saliva, and blood specimens along with stool, grossly normal colorectal mucosal biopsies, and cytology brushings collected during a flexible sigmoidoscopy. As biomarker endpoints, the effect of aspirin on urinary prostaglandin metabolites (PGE-M; primary endpoint), plasma inflammatory markers (macrophage inhibitory cytokine-1 (MIC-1)), colonic expression of transcription factor binding (transcription factor 7-like 2 (TCF7L2)), colonocyte gene expression, including hydroxyprostaglandin dehydrogenase 15-(NAD) (HPGD) as well as Wnt-signaling transcripts, colonic cellular nanocytology, and oral and gut microbial composition and function will be assessed. This study will help to further understand the impact of aspirin on CRC adenoma biology, and may provide insight into the role of platelets in the chemopreventive mechanisms of aspirin.

### The biochemistry of platelets

Platelets are derived from highly specialized precursor cells called megakaryocytes. These cells, found within the large bone marrow, function solely to produce and release platelets into the blood circulation. Once committed hematopoietic stem cells (HSC) encounter appropriate levels of cytokines to mature into platelet-forming megakaryocytes, they undergo a series of endomitotic cycles to replicate DNA without undergoing mitotic cell division. This process known as polyploidization is essential for the production of platelets and enables the controlled amplification of functional genes. Following endomitosis, the megakaryocyte maturation stage begins with enlargement of a cytoplasmic cavity and acquisition of platelet-specific proteins and organelles. This is followed by a cytoplasmic structural rearrangement aimed at platelet production which includes the development of a demarcation membrane system (DMS), assembly of a dense tubular network, and formation of granular platelet components. The attributed function of the DMS is to serve as a membrane reserve for proplatelet formation and extension. The dense tubular system also marks an area of prostaglandin biosynthesis and is likely to be encapsulated as pro-platelets begin to bud from the megakaryocyte membrane system. Finally, the progressive formation and appearance of a variety of secretory granules indicates that the megakaryocyte is primed to initiate the biogenesis of platelets.

Functional platelets are anucleated 2–5 μm disk-shaped cellular components, with a mean volume of 6–10 femtoliters. This small volume, approximately 6 orders of magnitude lower than a eukaryotic cell, contains all the growth factors, granules and organelles necessary for protein translation [65–67], post-translational modification [68], degradation of waste components [69, 70], and the necessary signaling components required to regulate cellular processes [71–74]. The messenger RNA (mRNA) content of platelets mirrors the mRNA content found in megakaryocytes [75–77] indicating that transcriptional production of platelet mRNA occurs exclusively in the megakaryocyte.

## The role of platelets in the clotting cascade

Platelets play a critical role in hemostasis, thrombosis, and maintenance of blood vessel integrity. Inadequate regulation in platelet activity can lead to inappropriate bleeding, whereas excessive activity leads to thrombosis and acute ischemic events. The clotting process is regulated by membrane surface glycoproteins and receptors that trigger the clotting cascade following binding by exogenous effectors such as adenosine diphosphate (ADP), thromboxane A_2_ (TXA_2_), serotonin, collagen, thrombin, and epinephrine. The basic sequence in platelet aggregation occurs in three steps: initiation, extension, and stabilization. At the initiation stage, platelets become tethered to exposed von Willebrand factor (vWF)/collagen complexes and remain at the location of vascular injury and response long enough to trigger further activation by collagen. Amplification is characterized by a second wave of secretion and aggregation and is further enhanced by platelet-mediated release of thrombin, adenosine diphosphate (ADP), and thromboxane A_2_ (TXA_2_). The second wave also marks the extension phase in which newly arriving platelets adhere to the initial platelet monolayer. Following protein receptor-effector interaction, activated platelets continue to aggregate forming bridges between surface glycoproteins, fibrinogen, fibrin, and vWF to activated glycoproteins. This stabilization phase includes subsequent signaling events in the platelet plug formation that allows consolidation of the platelet aggregate to prevent its dispersal by shear forces in the circulating blood.

**Fig. 3:**
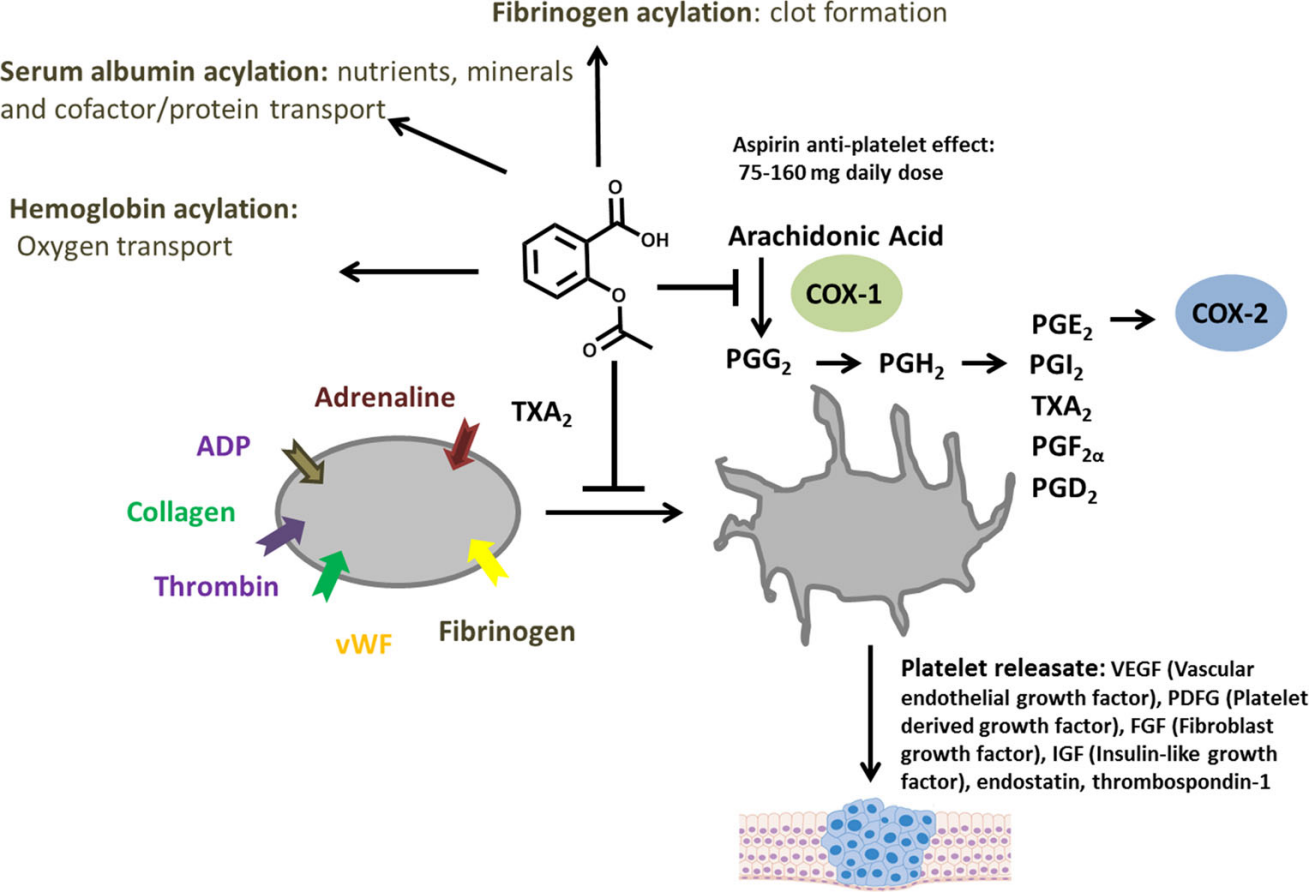
Platelet Activation. Platelet activation is initiated by multiple stimuli including thrombin, ADP, and fibrinogen. This results in the initiation of prostaglandin synthesis by COX-1 which is directly inhibited by aspirin. Aspirin can also modulate the clotting response by acetylating other serum proteins, most notably fibrinogen

Signaling in platelet aggregation begins with the activation of the receptors on the platelet surface by agonists such as collagen, thrombin, ADP, TXA_2_, and epinephrine. Activation of these GPCR receptors leads to the activation of phospholipase A2, which cleaves phosphotidyl choline and other membrane phospholipids, liberating arachidonate from the C2 position of the glycerol backbone. Arachidonate can be transformed into a variety of prostaglandins (PGD_2_, PGI_2_, PGE_2_, PGF_2α_,) that mediate the pro-inflammatory response. In addition, thromboxanes, such as TX_A2_, induce platelet release, aggregation and clotting, in addition to amplifying circulating platelet activation. Prostaglandin endoperoxide synthase-1 (PGSH-1)/cyclooxygenase-1 (COX-1) carries out the initial cyclooxygenase and peroxidase reactions to convert arachidonic acid to the precursor metabolites, prostaglandin G_2_ and prostaglandin H_2_. These are chemically elaborated to generate other prostaglandins, including TXA_2_, which mediates the majority of the platelet-derived response. A summary of the signaling events occurring during platelet activation is shown in Fig. 3.

### Aspirin modulates platelet activity and biology—cyclooxygenase inhibition

Aspirin inhibits platelet aggregation and prostaglandin release [78]. Early studies using aspirin radiolabeled with ^14^C at the acetate carbonyl carbon showed <0.1% of the ^14^C radiolabel was taken up by platelets and that the activity was associated predominantly with three proteins [39]. Although the association was irreversible, indicating the formation of a covalent bond, it was only found to be saturatable at biologically relevant concentrations for a single 85 kDa protein. The other two soluble platelet proteins showed non-saturatable acetylation suggesting that aspirin-dependent acetylation in platelets is both specific and non-specific. The acetylated 85 kDa enzyme was later found to be prostaglandin endoperoxide synthase-1 (PTGS-1/COX-1) [44]. Inhibition of platelet COX-1 by acetyl transfer is irreversible, and inhibition is maintained throughout the 10-day life of the platelet.

COX-1 is a bifunctional enzyme constitutively expressed in most tissues, and carries out two different and sequential reactions at spatially distinct, but mechanistically coupled active sites. In normal cells, COX-1 is membrane-bound and embedded in the luminal surface of the endoplasmic reticulum as well as the inner and outer surfaces of the nuclear envelope. Platelets, however, are anucleate cell fragments and instead express COX-1 proteins in the dense tubular membrane system that originates from the demarcate membrane system during platelet biogenesis. This dense tubular membrane system plays an important role in platelet activation, and is the principal site for eicosanoid synthesis in platelets [79, 80]. The 85 kDa COX-1 homodimer contains 576 residues and it is glycosylated at various lysine side chains. Although COX-1 is one of the few proteins that has been associated with aspirin inhibition at its active site, it is important to note that glycosylation of lysine residues enhances acetylation of other residues, and in this case, the serine at the active site of COX-1 [81]. Each COX-1 structural subunit incorporates three folding domains: an epidermal growth factor domain, a membrane-binding domain, and a catalytic active site. The catalytic domain contains a cyclooxygenase site that carries out the di-oxygenation of arachidonic acid to form a hydroxyl endoperoxide prostaglandin G_2_ (PGG_2_), while the adjacent peroxidase active site carries out the reduction of PGG_2_ to PGH_2_. Opposite to the membrane-active domain, within the catalytic domain, is the peroxidase active site which has a heme cofactor bound to a shallow cleft. The heme group is essential to the activation of a tyrosyl radical in the cyclooxygenase active site for lipid peroxidation of arachidonic acid. Opposite the heme-binding peroxidase site at the top of a tunnel originating in the membrane-binding domain is the cyclooxygenase active site. Arachidonic acid binds at this site, resulting in a repositioning the carboxylate of the substrate for di-oxygenation [37]. Structural studies of ovine COX-1 treated with 2-bromoacetoxy benzoic acid suggest that the binding of arachidonic acid to the cyclooxygenase end of the active site, and consequently the double oxygenation of arachidonic acid, are inhibited as a result of Serine 530 acetylation [37].

Irreversible inhibition of COX-1 by aspirin acetylation of the active site serine dramatically decreases prostaglandin biosynthesis. In platelets, COX-1 cannot be rapidly regenerated, and consequently COX-1 activity can only be recovered by new platelet biogenesis. Synthesis of thromboxane A2, prostaglandin E2 and prostacyclin (PGI_2_) are the most heavily affected in aspirin-treated platelets resulting in a deficiency in the clotting mechanism [82, 83], decreased secretion of gastric mucosa, increased irritation by gastric acids [84–86], as well as altered pathophysiological clotting, and vasodilation/constriction [82, 83, 87].

COX-2 is 60% identical to COX-1 at the amino acid level and their three-dimensional structures are nearly superimposable. COX-2 is inducible, and its expression is enhanced by the same prostaglandins that are synthesized by COX-1 in platelets and epithelial cells. COX-2 is overexpressed during megakaryocytopoeisis [88] and has been identified in the cross-sectional bone marrow samples from patients with chronic myeloid leukemia and polycythemia vera [89]. Another study characterized the levels of COX-2 expression in platelets in relation to COX-1, by directly measuring mRNA levels [90]. It was found that platelets express COX-2 at levels comparable to some malignant epithelial cells, albeit at significant lower levels than platelet COX-1. Acetylation of COX-2 in endothelial and epithelial cells inhibits biosynthesis of PGI_2_ and PGE_2_, which have different effects on downstream processes, such as inflammation. Although aspirin-mediated inhibition of COX-1 and COX-2 results in distinct profiles of prostaglandin biosynthesis inhibition, the basis for inhibition in both cases is the blockade of prostaglandin endoperoxide synthase and consequential reduction in multifunctional prostaglandin H_2_ levels. The role of COX-3 in the context of platelet biology remains unknown.

The COX-inhibitory activity of aspirin is contingent on the administered dose. Low doses, those ranging from 75 to 300 mg, result in selective inhibition in platelet TXA_2_ production without suppressing prostacyclin (PGI_2_), a common platelet antagonist and vasodilator. PGI_2_ is expected to be derived mainly from vascular COX-2 suggesting that COX-2 inhibition is minimal in the low-dose regime. Increased doses (>1200 mg) have analgesic and anti-inflammatory properties, properties associated with the pathophysiological inhibition of COX-1 and COX-2. It is important to note that COX-2 can also utilize arachidonic acid for synthesis of lipoxins, particularly 15-hydroxyeicosatetraenoic acid (15-HETE). This biosynthetic route is expected to remain intact even after COX-2 acetylation [91, 92]. This differential inhibition of COX activities can be explained, in part, by the relative inhibitory potency of aspirin. Although aspirin is typically thought of as a non-specific COX inhibitor, it is highly selective for COX-1 *versus* COX-2. As seen in Blanco et al. [93], the IC_50_ of aspirin for COX-1 is approximately 3.5 μM while the IC_50_ for COX-2 is approximately 30 μM. While the aspirin-reactive active sites of both enzymes are homologous, acetylation of Ser-516 of COX-2 results in only partial inhibition of catalytic activity [94, 95]. Given the achievable serum concentration in the low-dose regime (~7 μM), it is unlikely that the COX-2 is more than 5% acetylated while platelet-derived COX-1 is likely to be >70% acetylated [95]. This suggests that regular low-dose aspirin will invariably maintain COX-1 inhibition in circulating platelets, with minimal effect in the inhibition of peripheral COX-2. A summary of the effects of low-dose and high-dose aspirin on COX activity in blood and tissue is shown in Table 1.

**Table 1:** Effect of aspirin dosage (low dose <300 mg, high dose >650 mg) on various environments in the body

Platelet effect COX-1	Megakaryocyte COX-1/COX-2	Endothelial/stromal COX-2
Aspirin administration 75–150 mg	Aspirin administration >300 mg	Aspirin administration >300 mg
• Presystemic inhibition of COX-1	• Systemic inhibition of COX-1 and COX-2	• Pre-systemic inhibition of COX-1
• Complete suppression of TXA_2_ production	• Longlasting duration of TXA_2_ suppression	• Systemic inhibition of COX-1 and COX-2
• Effect cumulative upon repeated	• Residual effect of repeated doses	• Longlasting duration of TXA_2_ suppression

### Aspirin-dependent acetylation of platelet-interacting proteins in the blood

Platelets express a variety of surface receptors that allow them to interact with plasma and blood proteins, pathogens, pathogen-related products, and the inflamed endothelium. Surface receptors are critical to platelet adhesion to the injured vasculature, formation of the clotting thrombus, and activation *via* a number of metabolic effectors. The interaction between platelets and other blood proteins in the systemic circulation is critical for the execution and resolution of the clotting response. Interestingly, many of these proteins are also modified by aspirin.

#### Fibrinogen

Farr and co-workers identified fibrinogen in 1968 as a target of aspirin acetylation. Fibrinogen is found as a soluble protein in plasma as well as an intracellular membrane-associated protein in platelets [96–98]. Fibrinogen accounts for 3–10% of the total platelet protein (with close to 25% found in α-granules [98]) and is released upon platelet activation. Fibrinogen has been reported to be acetylated *in vitro* and *in vivo* by aspirin to form ε-N-acetyl lysine derivatives with an average of three residues of fibrinogen undergoing modification. Acetylated fibrinogen increases susceptibility of fibrin clots to lysis [99].

#### Albumin

Albumin modification by aspirin acetylation has been known for over half a century [22]. A number of studies from Farr and coworkers have assessed the possible conformational effects triggered by the acetyl group addition to albumin [22, 50]. The most discussed modification of serum albumin in the literature focuses on the acetylation of lysine residues [22, 50]. Human serum albumin has also been observed to affect platelet-clotting mechanisms by influencing calcium regulation [100].

#### Hemoglobin

Perhaps the most important component of the blood-plasma milieu, hemoglobin, undergoes aspirin-dependent acetylation *in vitro*, and it is presumed to undergo similar modifications at high aspirin doses *in vivo* [101]. Studies of hemoglobin acetylation by aspirin demonstrated decreases in protein glycation, and in the presence of high glucose concentrations hemoglobin acetylation by aspirin is increased [102], an effect that has also been observed in serum albumin. Aspirin is able to acetylate a variety of lysine residues in the α and β chains of hemoglobin, without having an effect on its structural conformation or oxygen binding and transport functions [40]. Hemoglobin is able to trigger platelet aggregation *via* interactions with GP1βα, one of the many platelet surface receptor proteins. Relatively low concentrations of hemoglobin are also capable of inducing platelet aggregation, although the effect of hemoglobin acetylation by aspirin on the interactions between hemoglobin and platelets remain unknown [103].

### Effect of aspirin on the platelet releasate: implications for cancer

Cyclooxygenase inhibition and the concurrent reduction in thromboxane biosynthesis result in reduced platelet aggregation, expression of P-selectin, and attenuated clotting function. In addition to its role in modulating platelet aggregation, aspirin has also been shown to alter the profile of expressed and secreted proteins in platelets. Many of these proteins are involved in mediating the clotting response and recruiting immune cells to the site of injury [104]. However, many proteins found in the platelet “releasate” can also play an important role in promoting angiogenesis and tumor growth.

Aspirin has been shown to inhibit the release of interleukin 7 (IL-7) by platelets stimulated with thrombin receptor activating peptide (SFLLRN). Healthy patients taking aspirin also showed significantly lower plasma IL-7 [105]. This pro-inflammatory cytokine has been shown to play a key role in both B cell and T cell maturation [106, 107]. IL-7 has also been shown to have both pro- and anti-tumor effects with the latter resulting mainly from inhibition of apoptosis through regulation of BCL2 [108]. Platelets are also a source of pro-angiogenic factors including VEGF and angiopoetin-1 and there is some evidence to suggest that regular aspirin use reduces the plasma concentration of both factors although it is unclear whether this is purely a function of platelet releasate [109]. This is supported by a clinical study in which aspirin therapy appeared to favor an overall antiangiogenic balance in women with breast cancer who received tamoxifen as assessed by decreasing plasma VEGF levels and thrombin receptor mediated release of TSP-1 and VEGF from platelets [110].

Coppinger et al. carried out a mass spectrometry-based proteomic study to further explore the composition of the platelet releasate as a function of aspirin treatment [111]. In this study, treatment of human platelets with low-dose aspirin (20 μM) following stimulation by collagen, SFLLRN, or ADP resulted in a broad decrease in the amount of protein found in the releasate although the extent of this reduction was dependent on the agonist used. Aspirin treatment was also found to result in decreased levels of growth regulating growth factor (GRO), platelet-derived growth factor (PDGF), angiogenin, RANTES, and oncostatin M (OSM) in the platelet releasate, particularly following stimulation with collagen. While these, and other platelet-derived cytokines (e.g., CXCL4 and CTGF [112–114]), are critical for regulating vascular repair, they also play a role in driving tumorigenesis, angiogenesis, and metastasis.

### Defining the aspirin acetylome

As stated above, aspirin is known to acetylate a wide variety of intracellular and extracellular protein targets, particularly at side chain and N-terminal amino groups. Unfortunately, there have been no comprehensive proteomic studies that specifically address the question of which platelet proteins, besides the COX enzymes, are acetylated by aspirin or the biological role of these non-canonical acetylations. In this section, we will consider previous proteomic studies to identify non-canonical targets of aspirin-mediated acetylation and attempt to relate them to the current state of platelet proteomics.

There have been numerous proteomic studies that have attempted to define the set of proteins acetylated by physiological concentrations of aspirin in various cell lines. Bhat and co-workers identified 33 cellular proteins acetylated by aspirin after enrichment with an anti-acetyl lysine antibody [115]. Subsequent analysis by mass spectrometry identified the presence of acetylated cytoskeletal and metabolic enzymes, including glucose-6-phosphate dehydrogenase (G6PD), lactate dehydrogenase, enolase, pyruvate kinase, and transketolase although only G6PD was significantly inhibited by aspirin-mediated acetylation *in vitro*. This suggests that aspirin may block flux through the pentose-phosphate pathway although additional studies are necessary to confirm this. This group also showed that aspirin acetylates p53 which results in enhanced DNA binding, expression of p21^Cip^, and enhancement of apoptotic cell death in the presence of camptothecin [30, 116]. While these effects have been demonstrated in multiple tumor cell lines, the absence of p53 in the platelet proteome [117] as well as the lack of a functional genome in platelets suggests that p53 acetylation will have minimal impact on platelet biology.

More recent advances in high-throughput proteomics coupled with activity-based probes have led to the identification of hundreds of putative aspirin-mediated acetylations. In one approach, the acetyl group of aspirin was replaced with pentynoic acid to generate an alkyne-containing aspirin derivative (AspAlk) [118]. In contrast to aspirin, acetyl transfer by AspAlk results in the incorporation of an azide-reactive alkyne at sites normally acetylated by aspirin. After incubation of AspAlk with live colorectal HCT-15 cells, proteins that were acetylated by AspAlk were tagged with biotin *via* the copper-catalyzed azide-alkyne cycloaddition (CuAAC) and isolated by streptavidin pulldown. Following analysis by LC-MS, the authors were able to identify 120 proteins with significant enrichment of AspAlk acetylation relative to DMSO controls. The most highly enriched classes of proteins in this study were those involved in protein synthesis and folding, cytoskeletal proteins, and metabolic enzymes. Histone acetylation was also observed and confirmed biochemically. This work was extended in a recent manuscript by Shen, Lin, and co-workers who used an acid-labile biotin azide to facilitate retrieval of AspAlk-modified proteins following biotin conjugation and streptavidin pulldown [119]. This strategy resulted in the identification of over 500 acetylated proteins. Pathway analysis of the target list indicated significant acetylation within the mTOR pathway which controls many key cellular functions including protein synthesis and autophagy. The authors validated the initial proteomic observations by showing that aspirin treatment of both HCT116 colorectal cells and mouse embryonic fibroblasts reduced *de novo* protein synthesis and induced autophagy. The induction of autophagy by aspirin is of particular interest in the light of a recent study showing that autophagy is essential for normal platelet function and is upregulated during platelet stimulation [120]. Furthermore, functional autophagic machinery is essential for platelet anti-coagulant activity as demonstrated by mouse models where platelet *Atg7* is knocked out. While the functional relationship between aspirin-mediated acetylation and platelet autophagy induction remains unclear, inhibition of the pentose phosphate pathway (PPP) through G6PD blockade or disruption of mitochondrial respiration through acetylation of malate dehydrogenase and/or isocitrate dehydrogenase may increase the intracellular oxidative burden, a known trigger for autophagy [121–123]. Alternatively, there is ample evidence of aspirin-mediated acetylation of chaperones, particularly heat shock proteins and peptidyl-prolyl isomerases [108] which may impair proper protein-folding and trigger autophagic removal of misfolded proteins.

Another recent proteomic study by Tatham and co-workers used ^3^H-labeled aspirin to identify sites of aspirin-mediated acetylation in HeLa cells by mass spectrometry [124]. In this approach, the use of tritiated aspirin results in a +3 Da mass shift relative to normal acetate and allows more accurate discrimination of aspirin-mediated acetylation *versus* endogenous acetylation. This approach revealed over 12,000 aspirin-mediated acetylations in over 3700 unique proteins. Interestingly, many of the proteins found to be acetylated by aspirin were also found to be acetylated in the absence of aspirin suggesting that aspirin “amplifies” existing protein acetylation sites. The authors of this study also found that in most cases, <1% of the total sites available for acetylation on any particular protein were acetylated by aspirin implying that the stoichiometry of non-specific aspirin-mediated acetylation may be insufficient to produce significant biological effects without pharmacologic blockade of endogenous deacetylase activities.

This study also showed significant acetylation of histone proteins with the majority of the aspirin-mediated acetylation occurring in the histone core rather than the N-terminal tails. Histone acetylation has been observed in multiple proteomic studies [118, 119] and is somewhat unsurprising given the high proportion of nucleophilic lysine residues in histones that play a significant role in electrostatic DNA binding. Histone acetylation plays a critical role in DNA binding and is a well-known epigenetic mechanism for regulating gene expression [125]. Although platelets are anucleate, previous transcriptomic studies have identified histone-specific transcripts in platelets, particularly H2A, H2B, H3, and H4 [125]. While histone acetylation by aspirin has been convincingly demonstrated, it is important to note that histone *expression* has not been confirmed in platelets. Rather, it has been postulated instead that the presence of histone transcripts in platelets is an artifact of aberrant cell cycling in megakaryocytes that give rise to platelets [126]. More importantly, the role of histones in controlling expression at the DNA level is absent in anucleate platelets.

### Effects of aspirin on platelet metabolism

Platelet metabolism is primarily oxidative in contrast to neutrophils which are primarily glycolytic [127, 128]. Blockade of anaerobic glycolysis does not decrease ATP nor does it inhibit platelet function. It has been shown that acetylation of tricarboxylic acid (TCA) cycle enzymes and electron transport chain (ETC) components is a common method of regulation particularly for enzymes involved in metabolism, such as malate dehydrogenase in carbon metabolism [129–131], the regulation of lipid metabolism [129, 132], and in the urea cycle for ammonia detoxification [129, 133]. It follows then that acetylation of TCA cycle enzymes and ETC components may have a significant effect on platelet bioenergetics. Proteomic studies of aspirin acetylation have consistently revealed aspirin-mediated acetylation of malate dehydrogenase, which regulates the switch between carbohydrate and fatty acid synthesis [134], and isocitrate dehydrogenase, which is regulated in the mitochondria by deacetylation through sirtuin (Sirt3 and Sirt5) proteins [135]. Sirtuin deacetylase activity is associated with a number of TCA enzymes in the mitochondrial matrix [135] and is believed to regulate antioxidant regeneration, TCA flux regulation, and anapleurosis [135]. While at the preparation and printing of this review, we are not aware of any studies that directly address the extent of aspirin-mediated acetylation of metabolic enzymes or the effect of aspirin on metabolic flux, the proteomic evidence suggests that this may be an important non-canonical effect of aspirin on platelet biochemistry.

### Effects of aspirin on platelet localization in tumors

New evidence is emerging that platelets themselves may play a significant role in carcinogenesis and more specifically in the development of metastasis. In mouse metastatic models where tumor cells are injected directly into the circulation, strategies to reduce the circulating platelet count have proved effective in reducing the tumor burden [136, 137]. Other studies in metastatic models where soluble fibrin and tumor cells were co-injected to enhance the clotting effect, showed increased incidence of metastasis *in vivo* [138, 139]. These studies were supported by *in vitro* experiments where soluble fibrin enhanced interactions between platelets and tumor cells in culture conditions [140]. These studies support the hypothesis that platelet aggregation activation, in addition to the expected increase in fibrin, increases platelet adhesion to tumor cells and facilitates metastatic spread. In addition to fibrin, additional studies have considered the role of thrombin, PAR-1, and coagulation factor VII (FVII) [141], and their association with enhancement in cancer cell viability [142], cancer growth and dissemination [143], increased tumor malignancy [142–144], and metastatic support [145].

In addition to modulating the biology of tumor cells in systemic circulation, platelets have also been shown to play an important role in the growth of tumor cells. In one study, it was shown that aspirin significantly decreased the degree of proliferation cells both *in vitro* and *in vivo* of ovarian cancer [146]. This same study also found that platelet activation can increase proliferation and tumor cell growth after co-incubation of tumor cells and platelets. Inhibition of platelet adhesive receptors including GPIβα, GPIIβIIIα, and P-selectin however did not diminish proliferative effects from platelets. This suggests that platelet-secreted proteins and other factors may play a role in regulating tumor cell growth. For example, it was observed that the reduction in platelet TGF-β1 decreased the proliferation of ovarian cancer cells exposed to platelets [146]. Furthermore, aspirin has also shown to prevent colorectal cancer metastasis through a COX-1 mechanism involving TXA2 and PGE2 [147], suggesting that activated platelets may support metastasis throught COX-1 dependent prostaglandin production. Finally, a novel aspirin-phosphotidylcholine conjugate (Aspirin-PC) has been shown to disrupt platelet-tumor cell induced epithelial-mesenchymal transition (EMT) through VEGF and thromboxane release. This formulation was also found to inhibit cell proliferation and angiogenesis while increasing apoptosis in ovarian and colorectal cancer cell models [148, 149].

## Conclusions and future directions

Aspirin-mediated acetylation of cyclooxygenase enzymes is believed to be the primary mechanism for its anti-inflammatory, anti-pyretic and anti-platelet effects. While the platelet-dependent effects of aspirin have been extensively studied in the context of COX-1, the role of non-canonical (e.g., non-COX) aspirin-mediated acetylation in platelets remains largely unknown. Numerous proteomic studies have attempted to identify the “off-target” effects of aspirin in both normal human and cancer cell lines, and such studies have proved valuable in understanding aspirin prophylactic effects.. However, there still remains a significant gap between identifying a target of acetylation *in vitro* and determining the extent and biological significance of this modification in platelets *in vivo*. In addition, we believe that it will be highly informative to extend proteomic studies of aspirin-mediated acetylation to platelets, particularly in light of recent work characterizing the platelet proteome. It would be of considerable interest to correlate the platelet acetylome with metabolic studies to determine the effect of aspirin treatment on platelet metabolism, particularly given the emerging role of metabolism in carcinogenesis and development of metastasis. As our understanding of the cross-talk between platelets, tumor cells, and the immune system expands, the COX-dependent and COX-independent effects of aspirin in modulating interactions at the biochemical level can now be directly addressed.
